# Impact of Sarcopenia on Clinical Outcomes of Minimally Invasive Lumbar Decompression Surgery

**DOI:** 10.1038/s41598-019-53053-0

**Published:** 2019-11-12

**Authors:** Hiromitsu Toyoda, Masatoshi Hoshino, Shoichiro Ohyama, Hidetomi Terai, Akinobu Suzuki, Kentaro Yamada, Shinji Takahashi, Kazunori Hayashi, Koji Tamai, Yusuke Hori, Hiroaki Nakamura

**Affiliations:** Department of Orthopaedic Surgery, Osaka City University Graduate School of Medicine, Osaka, Japan

**Keywords:** Ageing, Outcomes research

## Abstract

The purpose of this study was to clarify the clinical impact of sarcopenia on the outcome of minimally invasive lumbar decompression surgery. The records of 130 patients who were >65 years and underwent minimally invasive lumbar decompression surgery were retrospectively reviewed. We collected the Japanese Orthopaedic Association (JOA) score before surgery and at the final follow-up and measured appendicular muscle mass using bioimpedance analysis, hand-grip strength and gait speed. We diagnosed the patients with sarcopenia, dynapenia and normal stages using the European Working Group on Sarcopenia in Older People definition and used cutoff thresholds according to the algorithm set by the Asian Working Group for Sarcopenia. The average age of patients undergoing surgery was 76.9 years old. The JOA score improved from 12.6 points preoperatively to 24.3 points at final follow up. The prevalence of the sarcopenia, dynapenia and normal stages was 20.0, 31.6 and 43.8%. Clinical outcomes, such as JOA score, JOA score improvement ratio, visual analog scale for low back pain, leg pain and numbness, were not significantly different among each group. Multiple regression analysis showed that preoperative JOA score and low physical performance (low gait speed) were independently associated with poor clinical outcomes. The JOA score improved after minimally invasive lumbar decompression surgery even when the patients were diagnosed as being at different stages of sarcopenia. Low physical performance had the greater clinical impact on the clinical outcome of lumbar surgery than low skeletal muscle index.

## Introduction

According to the United Nations, the number of older persons worldwide is expected to double between 2017 and 2050. It was reported that one in eight people were aged 60 or over in 2017 and one in five will be aged 60 or over in 2050^1^. As older people age, their physical and mental functions gradually decline, the levels of activity and independence in daily living decrease, and nursing care becomes necessary. Sarcopenia and frailty have been recognized as major public health issues because they are both easily detectable clinically and relatively simple to prevent or treat^2,3^. Identification of frailty and sarcopenia in its early stages is important for implementing early treatment and intervention.

In 2010, the European Working Group on Sarcopenia in Older People (EWGSOP) proposed an operational definition and diagnostic strategy for sarcopenia^4^, and it has become widely used in the world. The EWGSOP recommends using the presence of low muscle mass, low muscle strength and low physical performance to diagnose sarcopenia. According to their conceptual staging, low muscle strength was affected by low muscle mass, and low physical performance was affected by low muscle strength. However, the relationship between muscle mass and strength is reportedly no-linear^5^. Therefore, it is thought to be limited clinical value to define sarcopenia only in terms of muscle mass. The term dynapenia was defined to describe age-related loss of muscle strength and function. Dynapenia is also one risk factor of mobility limitations and mortality^6^. In recent years, we can easily classify the stage of sarcopenia and dynapenia using the Asian Working Group for Sarcopenia (AWGS), which developed the strategy for sarcopenia screening and cutoff values for assessment^7^. We have performed a cross-sectional study to evaluate the incidence of sarcopenia or dynapenia in outpatient clinic for patient with spinal disorders and described that the respective incidences of the sarcopenia, dynapenia, and normal stages were 16.4%, 26.7%, and 56.9% for males, and 23.7%, 50.9%, and 25.4% for females^8^.We have also reported that dynapenia was more prevalent than sarcopenia in patients with spinal disorders. It is quite difficult to discriminate between the age-related changes in neurophysiology and neurologic diseases caused spinal diseases, and the relationship between sarcopenia, dynapenia and clinical outcomes of spinal surgery remain unknown. Therefore, in the present study we focused on lumbar spinal stenosis (LSS) and investigated the clinical impact of sarcopenia on the outcome of minimally invasive lumbar decompression surgery.

## Methods

### Study population

From August 2015 to July 2016, a total of 230 consecutive patients who visited our spinal outpatient clinic were enrolled in the present cross-sectional observational study^8^. The study was approved by the ethics committee of Osaka City University Graduate School of Medicine (approval no. 3170), and all participants provided written informed consent prior to enrollment. The research was done in accordance with the Declaration of Helsinki. We excluded the patients who had any metal implants or internal electrical device in the body and were not able to stand by themselves. Of the 230 eligible patients, the records of 130 patients who underwent minimally invasive lumbar decompression surgery were reviewed for this study.

### Surgical procedures

All patients underwent bilateral decompression via a unilateral approach to decompress the central and bilateral lateral recess using a microscope or the METRx Microendoscopic Discectomy System (Medtronic Sofamor Danek, Warsaw, Indiana, USA), performed as previously described^9,10^. The radiological indications were LSS, degenerative lumbar spondylolisthesis (DS) with a Meyerding grade ≤1 and a posterior opening ≤5° during anterior flexion of the affected intervertebral level and degenerative lumbar scoliosis (DLS) with a Cobb’s angle ≥10° or ≤20°. Laminotomy was performed on the side of the approach in the area of the ligamentum flavum insertion, and resection of the articular process was performed in a trumpeted manner until the inner aspect of the pedicle, with slight tilting of the microscope or tubular retractor laterally. Laminotomy was performed on the approach side using an air drill, Kerrison rongeur and an osteotome. Decompression was then performed on the contralateral side after tilting the operating table and the microscope or the tubular retractor.

### Clinical outcomes

Clinical outcomes were evaluated with the Japanese Orthopaedic Association score (JOA score), visual analog scale (VAS) score for lower back pain, leg pain, and leg numbness preoperatively and at the latest follow-up. The improvement rate for the JOA scores was calculated as (postoperative JOA score−preoperative JOA score)/(29−preoperative JOA score) × 100 (%)^11^. We defined poor clinical outcomes as the bottom 25th percentile of the JOA score at the final follow-up.

### Measurements of sarcopenia related parameters

Muscle mass were assessed by BIA using a Tanita MC-980A Body Composition Analyzer (Tanita, Tokyo, Japan). It was reported that the muscle mass assessed by the BIA method shows a high correlation with the muscle mass assessed by the DXA method^12–14^. Skeletal muscle mass index (SMI) was defined by dividing appendicular skeletal muscle mass by height in meters squared (kg/m^2^).

Handgrip strength was measured using a T.K.K.5401 dynamometer (Takei, Niigata City, Japan). Both hands were tested and the best performance was used for the analysis.

The time taken to walk middle 5-m course (i.e. between the 2- and 7-m marks) at normal walking speed was recorded and the usual gait speed was calculated. The first and last meters were ignored to allow for acceleration and deceleration.

### Definition of sarcopenia

According to the AWGS algorithm, low skeletal muscle index (SMI) were <7.0 kg/m^2^ for men and <5.7 kg/m^2^ for women was used to defined the cutoff thresholds for sarcopenia in this study. Furthermore, the cutoff thresholds for low handgrip strength were <26 kg for men and <18 kg for women, and for low gait speed was <0.8 m/s^7^. In accordance with the EWGSOP consensus^4^, presarcopenia was defined as having only low skeletal muscle index, whereas sarcopenia was defined as low skeletal muscle index plus low handgrip strength or low walking speed. Furthermore, we defined severe sarcopenia as low skeletal muscle index plus low handgrip strength and low walking speed. Dynapenia was defined as low handgrip strength or low walking speed without low skeletal muscle index^5^. Using this definition of sarcopenia, we classified the subjects into sarcopenia, dynapenia and normal stage.

### Statistical analysis

All data are shown as the mean ± standard deviation. SPSS ver. 19.0 (SPSS Inc., Chicago, IL, USA) was used for statistical analyses. Comparison of the three groups was performed using the parametric one-way ANOVA, followed by multiple comparison tests using the Bonferroni method when a significant value was found. Because Group A was tested against Group B and C only, the p-value was adjusted to 0.025. The Kruskal–Wallis test followed by the multiple comparison test was used for nonparametric variables. Pearson’s correlation coefficient and simple regression analysis were used for analyzing the correlations between variables. The strength of the correlation was classified as very strong (≥0.80), strong (0.60–0.79), moderate (0.40–0.59), weak (0.20–0.39), or very weak (<0.20)^15^. To elucidate factors predicting poor clinical outcomes, multivariate statistical analysis was performed. Poor clinical outcome was defined as an outcome measure and explanatory variables including age, sex, JOA score before surgery, and sarcopenia related parameters (low muscle mass; low SMI, low muscle strength; low handgrip strength, low physical performance; low walking speed). Differences were considered significant at p < 0.05.

## Results

### Characteristics of the participants

There were 70 men and 60 women. The mean age of the patients at the time of surgery was 76.9 $\pm 6.4$ y old. Preoperative diagnoses were LSS in 102 patients, DS in 22 patients and LSS combined with disc herniation in 6 patients. Single-level, two-level and three-level decompressions were performed in 81, 35 and 14 patients, respectively. Microsurgery was performed in 61 patients and microscopic surgery was performed in 69 patients. The average follow-up period was 41.0 months.

Level of surgery was L2–3 in 18 discs; L3-4 in 108 discs; L4-5 in 181 discs; and L5-S1 in 12 discs. There were no persistent complications during or after the surgeries. The average JOA score improved 12.6 ± 4.3 points before surgery to 24.3 ± 3.6 points at the final follow-up. The average improvement rate of JOA scores (Hirabayashi’s improvement ratio) was 70.4 ± 20.3%.

### Comparison of baseline characteristics and clinical outcomes among sarcopenia, dynapenia and normal groups

**Figure 1:**
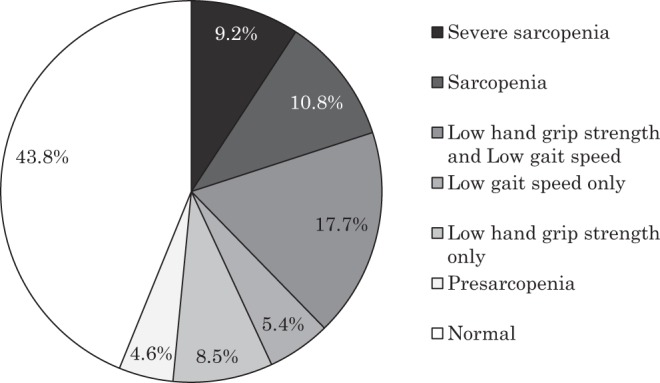
Prevalence of sarcopenia in patients with lumbar spinal stenosis after minimally invasive lumbar decompression surgery.

The prevalence of the sarcopenia, dynapenia and normal groups was 20.0%, 31.6% and 43.8%. Moreover, the prevalence of severe and pre-sarcopenia was 9.2% and 4.6% (Fig. 1). Table 1 shows the comparison of clinical outcomes of the sarcopenia, dynapenia and normal groups. The age of the sarcopenia group was significantly higher than that of the other groups (vs. dynapenia group; p = 0.047, vs. normal group; p < 0.001) and the prevalence of women in the dynapenia group and that of men in the normal group were significantly higher than the other groups (p < 0.001, p < 0.001). BMI and SMI in the sarcopenia group was significantly lower than those of the normal group (p < 0.001, p < 0.001). However, clinical outcomes, such as the JOA score, JOA score improvement ratio, VAS for low back pain (LBP), leg pain and numbness, were not significantly different among groups. The JOA score improved after minimally invasive lumbar decompression surgery even when the patients were diagnosed as being in the sarcopenia stage.

**Table 1:** Comparison of baseline characteristics and clinical outcomes between the sarcopenia, dynapenia and normal groups.

Variables	Sarcopenia group	Dynapenia group	Normal group	P value
Numbers (%)	26 (20.0)	41 (31.5)	57 (43.8)	
Age (years)	80.9 (5.7)	77.5 (6.3)	74.6 (5.8)	<0.001
Men/women	10/16	15/26	43/14	<0.001
BMI (Kg/m^2^)	19.9 (1.6)	23.8 (3.5)	24.6 (2.5)	<0.001
Follow up period (days)	1196.1 (977.8)	1330.3 (1216.3)	1093.8 (935.5)	0.573
SMI (kg/m^2^)	5.88 (0.66)	7.11 (1.12)	7.80 (1.07)	<0.001
Handgrip strength (kg)	20.3 (8.0)	22.1 (8.8)	23.2 (9.0)	0.374
Gait speed (m/s)	0.88 (0.23)	0.90 (0.27)	0.97 (0.29)	0.263
**JOA score**				
before surgery at the final F/U	12.8 (4.6) 24.0 (4.6)	12.8 (4.9) 24.0 (3.9)	12.2 (3.7) 24.4 (3.0)	0.737 0.826
Improvement ratio of JOA score (%)	66.8 (23.5)	68.9 (22.1)	72.9 (17.4)	0.447
**Low back pain**				
before surgery at the final F/U	43.5 (29.0) 29.8 (25.5)	44.2 (28.0) 19.6 (24.7)	48.2 (30.4) 19.1 20.8)	0.742 0.125
**Leg pain**				
before surgery at the final F/U	59.7 (27.9) 23.1 (29.0)	58.5 (29.7) 18.9 (29.6)	63.6 (27.0) 23.3 (27.4)	0.681 0.729
**Leg numbness**				
before surgery at the final F/U	56.1 (27.1) 26.5 (27.1)	56.8 (31.4) 39.3 (33.7)	60.0 (30.0) 27.4 (29.8)	0.474 0.126

### Relationships between the JOA score and sarcopenia related parameters

**Figure 2:**
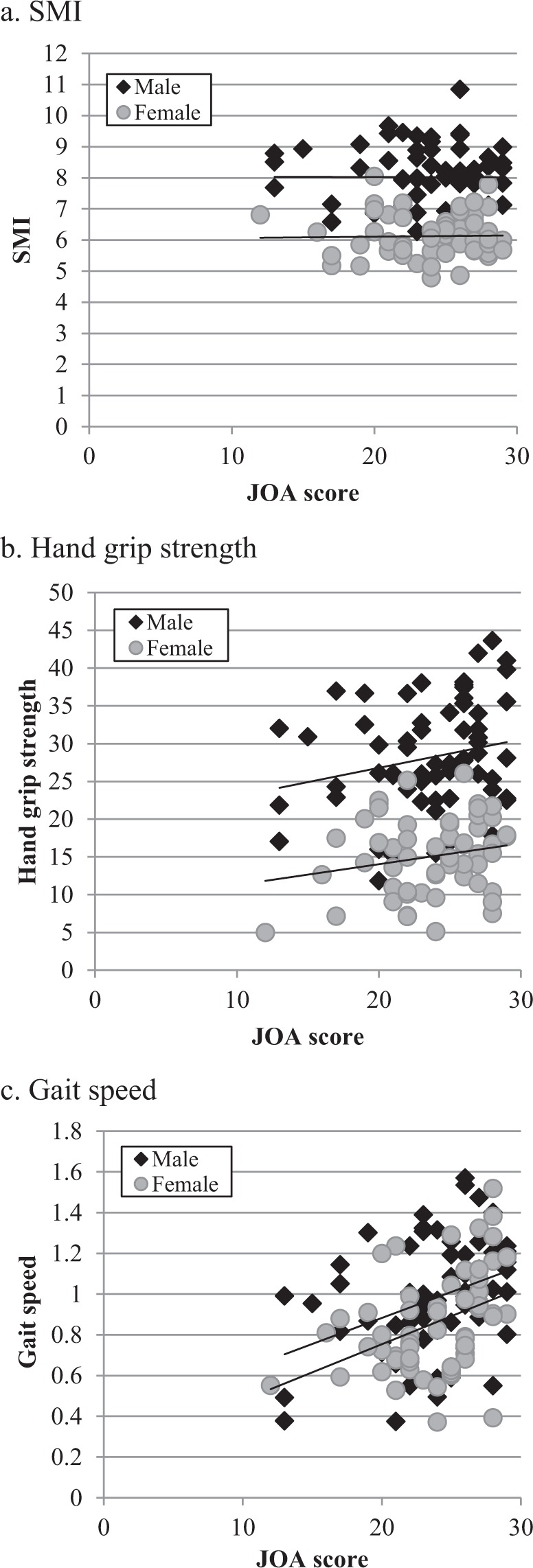
Correlation of the JOA score and SMI, handgrip strength and gait speed.

The correlations between the JOA score at final follow-up and age, JOA score before surgery, SMI, handgrip strength, and gait speed are shown in Table 2 and Fig. 2. Age, SMI and handgrip strength were not significantly correlated with JOA score at the final follow-up; however, the JOA score before surgery had a weakly significant correlation with the JOA score at final follow-up (r = 0.2, P = 0.03). Gait speed had a moderately significant correlation with JOA score at the final follow-up (r = 0.4, P < 0.001).

**Table 2:** Pearson correlation and p-value of the JOA score at final follow-up versus other parameters.

	Correlation	P value
Age	−0.087	0.328
JOA score before surgery	0.207	0.026
SMI (kg/m^2^)	0.016	0.859
Handgrip strength (kg)	0.121	0.175
Gait speed (m/s)	0.409	<0.001

To identify potential risk factors of poor clinical outcomes, we calculated the bottom 25^th^ percentile of the JOA score at the final follow-up. The cut-off value was 21 points and we used it as a dependent variable for multiple regression analysis. Multiple regression analysis showed that the JOA score before surgery and low physical performance (low gait speed) were independently associated with poor clinical outcomes (Table 3).

**Table 3:** Risk factors for poor clinical outcomes after minimally invasive lumbar decompression surgery.

Explanatory variable	Odds ratio	95% CI	P value
Age	1.04	0.96–1.13	0.321
Gender (Female)	1.39	0.48–4.00	0.546
JOA score before surgery	1.11	0.98–1.27	0.085
**Stage of sarcopenia**			
Low muscle mass	1.99	0.65–6.10	0.228
Low muscle strength	1.49	0.20–2.26	0.360
Low physical performance	5.15	1.64–16.12	0.005

## Discussion

LSS is common in older adult patients and is characterized by neurogenic claudication and such patients demonstrate walking intolerance and various physical disabilities. It was reported that LSS was the most common reason for spinal surgery in patients >65 years old^16^. The term sarcopenia was first described as the loss of muscle mass and performance associated with aging, although recently, recognized causes of sarcopenia also include malnutrition, physical inactivity, organ failure, invasive interventions, malignancy and other chronic disease. Physical inactivity plays a key role in the progression of sarcopenia and dynapenia. From these points of view, we investigated the prevalence of sarcopenia and dynapenia in patients with symptomatic LSS and examined whether the clinical outcome of LSS with sarcopenia could improve with surgery. Although several studies have investigated the prevalence of sarcopenia in healthy subjects^17^, no detailed data, as defined by AWGS, have been reported in patients with LSS. Therefore, we performed a cross-sectional observational study and revealed that the prevalence of sarcopenia and dynapenia in symptomatic LSS patients were 20.0% and 31.6%. The incidence of sarcopenia has also been reported to be 6–12% in studies with large sample sizes of more than 1000 participants^18–20^ and 7.5–8.2% in a Japanese population-based cross-sectional survey^21,22^. When compared with the previous reports, the prevalence of sarcopenia in the patients with symptomatic LSS might be greater than that of healthy subjects. We also compared the surgical outcomes of LSS with or without sarcopenia; however, the clinical outcomes, such as the JOA score, JOA score improvement ratio, VAS for LBP, leg pain and numbness, were not significantly different. Our finding indicates that the JOA score will improve after minimally invasive lumbar decompression surgery even when patients are diagnosed as being in a sarcopenia stage.

There are few reports describing the relationship between LSS and sarcopenia. Park *et al*. performed case-control study to investigate the prevalence of sarcopenia in patients with LSS and a matched control group^23^. They reported that sarcopenia was significantly higher in the LSS group (24%) (mean age, 67.9 y) when compared with the age- and sex-matched control group (12%) (mean age, 68.3 y). In their study, they did not use parameters of physical performance such as gait speed because they thought that deteriorated nerve function of the lower extremities influenced physical performance and/or muscle mass. They used only SMI plus the handgrip test determined by the AWGS and used only handgrip strength for within-group analysis for the implication of sarcopenia. Eguchi *et al*. reported that the prevalence of sarcopenia in female LSS (mean age, 74.4 y) was 9/34 (26.5%)^24^. They used only SMI for defining sarcopenia and used the diagnostic value (<5.46 kg/m^2^ for woman) determined by Sanada *et al*.^22^. Thus, prevalence depends substantially on the classification criteria and screening methods used. In the present study we used the guideline of the AWGS for diagnosis of sarcopenia. In accordance with the AWGS guidelines, sarcopenia was defined as low SMI (<7.0 kg/m^2^ for men, <5.7 kg/m^2^ for women), plus low handgrip strength (<26 kg for males, <18 kg for females) or low gait speed (<0.8 m/s). When researchers use only SMI plus handgrip strength or only SMI for diagnosis, the prevalence rate tends to increase because the samples with presarcopenia are included. If researchers use the lower cut-off value for diagnosis, the prevalence rate tends to decrease. In the present study, presarcopenia was also observed in 4.6% of patients; therefore, the prevalence rate of low SMI was increased to 24.6% (mean age, 76.9 y) and the female prevalence rate of low SMI was 33.3% (mean age, 77.2 y). Although it was quite difficult to determine the true values, we consider our results reasonable.

Sarcopenia is also thought to exacerbate lumbar spine disease; however, its specific impact on clinical outcomes following lumbar surgery remains controversial. Park *et al*. evaluated the sit-to-stand test, timed up and go (TUG) test, and clinical outcomes, including the Oswestry Disability Index (ODI) scores and the EuroQol (EQ-5D) to analyze the clinical impact of sarcopenia^23^. They reported that only the TUG test was significantly inferior in those with sarcopenia in the control group and the TUG test and ODI were significantly inferior in those with sarcopenia in the LSS group. Their results indicated the negative influence of sarcopenia on disability from LBP and balance and walking ability in the LSS group. Eguchi *et al*. reported that participants with sarcopenia scored significantly higher on the Roland–Morris Disability Questionnaire (RDQ) than normal participants; however, there were no correlations between other clinical outcomes such as VAS scores for LBP, JOA scores and SMI^24^. Following these previous reports and our results, it was speculated that accompanying sarcopenia may affect LBP-related activity of daily life or walking ability rather than JOA score and VAS that related to LSS. In the current study, 12 patients were diagnosed with severe sarcopenia. The mean age of the severe sarcopenia group was significantly higher than non severe sarcopenia group (76.2 ± 6.2 vs 83.6 ± 3.8 P < 0.001); however, the JOA score, VAS for LBP, leg pain and leg numbness were not significantly different between the two groups. Arinzon *et al*. reported that age was not a contraindication for surgical decompression of LSS, and Kim *et al*. reported that older adult patients who underwent spine surgery for spinal stenosis had mortality rates that were as good as or better than the corresponding general population^25,26^. Minimally invasive lumbar decompression surgery for LSS is a justifiable procedure not only in older adult patients but also for those diagnosed as having sarcopenia.

Despite the clinical outcomes such as the JOA score being not significantly different among each group, low physical performance (low gait speed) was independently associated with poor clinical outcomes. Gait speed has been described as the “sixth vital sign” with the potential to serve as a core indicator of health and function in aging and disease^27,28^. Performance measures have recently been identified as an important outcome for the LSS population^29^. Bohannon *et al*. reported that mean comfortable gait speed of adults aged between 20 and 79 y for men and women ranged between 1.33 and 1.46 m/s and 1.27 and 1.41 m/s^30^. Sun J *et al*. reported the comfortable speed results for men and women were 1.20 ± 0.13 m/s and 1.09 ± 0.09 m/s for normal subjects, and 0.99 ± 0.18 m/s and 1.01 ± 0.24 m/s for LSS patients^31^. Conrad *et al*. reported that the velocity of LSS patients was 1.01 ± 0.33 m/s for men and 0.75 ± 0.24 m/s for women^32^. In the present study, the gait speed results were 0.88 ± 0.23, 0.90 ± 0.27 and 0.97 ± 0.29 m/s for LSS patients with sarcopenia, LSS patients with dynapenia and w/o sarcopenia or LSS patients with dynapenia, respectively. These results suggest that gait speed in LSS patients was lower than that in healthy people regardless of sarcopenia stage. It is clear that walking is critical to overall health; therefore, a multidisciplinary assessment and increasing the overall level of physical activity in LSS may result in better outcomes for LSS.

There were several limitations to this review. (1) Because the present study was cross-sectional and did not include data from healthy subjects, we only compared our data with previous reports. The present results do not establish cause–effect relationships between sarcopenia/dynapenia and LSS. (2) The number of subjects included was small and postoperative follow-up periods were short. Our findings need to be confirmed in a larger population with longer postoperative follow-up periods. (3) The present study included only LSS patients who underwent minimally invasive lumbar decompression surgery. In the present study, the clinical outcomes were not significantly different among the sarcopenia, dynapenia and normal groups; however, the impact of sarcopenia on the clinical outcomes of open surgery or fusion surgery might be different. (4) Although BIA is widely used for measuring muscle mass, it has a tendency to overestimate muscle mass compared with DXA^33^. However, the BIA method reportedly shows a high correlation with the muscle mass obtained by the DXA method^12–14^. (5) In the present study, we indicated that low gait speed was independently associated with poor clinical outcomes; however, it is still unknown whether slow gait speed would be modestly improved after exercise or surgical intervention. In a future study, we want clarify the impact of surgery or excise on the LSS patient with sarcopenia.

## Conclusion

We investigated the prevalence of sarcopenia in patients with LSS using the consensus of the AWGS. The JOA score improved after minimally invasive lumbar decompression surgery even when patients were diagnosed as being in the sarcopenia stage. Low physical performance has a greater clinical impact on the clinical outcome of lumbar surgery than low skeletal muscle.

## References

[1] World Population Ageing 2017 Highlights, <http://www.un.org/en/development/desa/population/publications/pdf/ageing/WPA2017_Highlights.pdf> (2017).

[2] Beaudart C, Rizzoli R, Bruyere O, Reginster JY, Biver E (2014). Sarcopenia: burden and challenges for public health. Archives of public health = Archives belges de sante publique.

[3] Buckinx F (2015). Burden of frailty in the elderly population: perspectives for a public health challenge. Archives of public health = Archives belges de sante publique.

[4] Cruz-Jentoft AJ (2010). Sarcopenia: European consensus on definition and diagnosis: Report of the European Working Group on Sarcopenia in Older People. Age and ageing.

[5] Clark BC, Manini TM (2008). Sarcopenia =/= dynapenia. J Gerontol A Biol Sci Med Sci.

[6] Clark, B. C. & Manini, T. M. What is dynapenia? Nutrition (Burbank, Los Angeles County, Calif 28, 495–503. 10.1016/j.nut.2011.12.002 PMC3571692 22469110

[7] Chen LK (2014). Sarcopenia in Asia: consensus report of the Asian Working Group for Sarcopenia. J Am Med Dir Assoc.

[8] Toyoda, H. et al. The association of back muscle strength and sarcopenia-related parameters in the patients with spinal disorders. *Eur Spine J* (2018). 10.1007/s00586-018-5858-8 30542935

[9] Toyoda H (2011). Clinical outcome of microsurgical bilateral decompression via unilateral approach for lumbar canal stenosis: minimum five-year follow-up. Spine.

[10] Dohzono S (2015). The influence of preoperative spinal sagittal balance on clinical outcomes after microendoscopic laminotomy in patients with lumbar spinal canal stenosis. Journal of neurosurgery.

[11] Hirabayashi K, Miyakawa J, Satomi K, Maruyama T, Wakano K (1981). Operative results and postoperative progression of ossification among patients with ossification of cervical posterior longitudinal ligament. Spine.

[12] Yoshida D (2014). Development of an equation for estimating appendicular skeletal muscle mass in Japanese older adults using bioelectrical impedance analysis. Geriatr Gerontol Int.

[13] Kim M, Shinkai S, Murayama H, Mori S (2015). Comparison of segmental multifrequency bioelectrical impedance analysis with dual-energy X-ray absorptiometry for the assessment of body composition in a community-dwelling older population. Geriatr Gerontol Int.

[14] Fujimoto K (2018). Use of Bioelectrical Impedance Analysis for the Measurement of Appendicular Skeletal Muscle Mass/Whole Fat Mass and Its Relevance in Assessing Osteoporosis among Patients with Low Back. Pain: A Comparative Analysis Using Dual X-ray Absorptiometry. Asian spine journal.

[15] Evans, J. D. Straightforward statistics for the behavioral sciences. (Pacific Grove, 1996).

[16] Deyo RA (2010). Trends, major medical complications, and charges associated with surgery for lumbar spinal stenosis in older adults. Jama.

[17] Cruz-Jentoft AJ (2014). Prevalence of and interventions for sarcopenia in ageing adults: a systematic review. Report of the International Sarcopenia Initiative (EWGSOP and IWGS). Age and ageing.

[18] Janssen I, Heymsfield SB, Ross R (2002). Low relative skeletal muscle mass (sarcopenia) in older persons is associated with functional impairment and physical disability. J Am Geriatr Soc.

[19] Yoshida D (2014). Using two different algorithms to determine the prevalence of sarcopenia. Geriatr Gerontol Int.

[20] Castillo EM (2003). Sarcopenia in elderly men and women: the Rancho Bernardo study. Am J Prev Med.

[21] Yoshimura N (2017). Is osteoporosis a predictor for future sarcopenia or vice versa? Four-year observations between the second and third ROAD study surveys. Osteoporos Int.

[22] Sanada K (2010). A cross-sectional study of sarcopenia in Japanese men and women: reference values and association with cardiovascular risk factors. Eur J Appl Physiol.

[23] Park S (2016). The prevalence and impact of sarcopenia on degenerative lumbar spinal stenosis. The bone & joint journal.

[24] Eguchi Y (2018). Influence of Skeletal Muscle Mass and Spinal Alignment on Surgical Outcomes for Lumbar Spinal Stenosis. Asian spine journal.

[25] Arinzon ZH (2003). Surgical management of spinal stenosis: a comparison of immediate and long term outcome in two geriatric patient populations. Archives of gerontology and geriatrics.

[26] Kim, H. J. et al. Life expectancy after lumbar spine surgery: one- to eleven-year follow-up of 1015 patients. *Spine* **33**, 2116–2121, discussion 2122–2113 (2008). 10.1097/BRS.0b013e31817e1022 18758368

[27] Fritz S, Lusardi M (2009). White paper: “walking speed: the sixth vital sign”. Journal of geriatric physical therapy (2001).

[28] Studenski S (2009). Bradypedia: is gait speed ready for clinical use? The journal of nutrition, health &. aging.

[29] Conway J, Tomkins CC, Haig AJ (2011). Walking assessment in people with lumbar spinal stenosis: capacity, performance, and self-report measures. Spine J.

[30] Bohannon RW (1997). Comfortable and maximum walking speed of adults aged 20-79 years: reference values and determinants. Age and ageing.

[31] Sun J (2018). Clinical Gait Evaluation of Patients with Lumbar Spine Stenosis. Orthopaedic surgery.

[32] Conrad BP (2013). Associations of self-report measures with gait, range of motion and proprioception in patients with lumbar spinal stenosis. Gait & posture.

[33] Pietrobelli A, Rubiano F, St-Onge MP, Heymsfield SB (2004). New bioimpedance analysis system: improved phenotyping with whole-body analysis. European journal of clinical nutrition.

